# Vitamins as modulators of neonatal intestinal injury

**DOI:** 10.3389/fnut.2026.1860890

**Published:** 2026-07-01

**Authors:** Dhirendra K. Singh, Yukihiro Yamaguchi, Chieko Saito, Olivia G. Cassidy, Keita Nishiyama, Lei Huang

**Affiliations:** 1 Department of Rheumatology and Inflammation Research, University of Gothenburg, Gothenburg, Sweden; 2 Department of Pediatrics, University of North Carolina at Chapel Hill, Chapel Hill, NC, United States; 3 Department of Biology, American University, Washington, DC, United States; 4 Warren Alpert Medical School of Brown University, Providence, RI, United States; 5 Laboratory of Animal Food Function, School of Agricultural Science, Tohoku University, Sendai, Japan; 6 Faculty of Medical Sciences, Newcastle University, Newcastle upon Tyne, United Kingdom

**Keywords:** IgA, LPS, Th17, tight junction, TLR4, Treg, vitamins

## Abstract

Necrotizing enterocolitis (NEC) is a severe gastrointestinal disease that primarily affects premature infants. It is characterized by intestinal injury and tissue necrosis involving both small and large intestines. Loss of epithelial barrier integrity allows enteric bacteria to translocate into the bloodstream, frequently leading to severe sepsis and high mortality, with reported rates ranging from 15 to 45%. Infants who survive often experience long-term complications, including neurodevelopmental impairment, growth restriction, and short-bowel syndrome. Vitamins help maintain intestinal barrier integrity and regulate immune and inflammatory responses, suggesting an important role in the development and progression of NEC. Vitamins are important modulators of NEC pathophysiology and may serve as adjunctive therapeutic agents through multiple molecular mechanisms. Vitamins A and D promote epithelial barrier maturation and immune homeostasis by limiting excessive Toll-like receptor 4 (TLR4) activation, a major contributor to epithelial injury and inflammation in the immature intestine. Vitamins E and C reduce oxidative stress, a key feature of NEC, while supporting regulatory T-cell function and IgA-mediated mucosal immunity. However, their use in preterm infants requires careful consideration of dosage and route of administration. Vitamin K contributes to both immune regulation and hemostasis by maintaining protein S-dependent anticoagulant activity and suppressing TLR-mediated inflammatory signaling, thereby reducing intestinal microvascular injury. Folate attenuates lipopolysaccharide-induced inflammatory responses and may strengthen host defenses against Gram-negative bacterial stimuli implicated in NEC pathogenesis. Collectively, these vitamins target key pathways involved in NEC, including TLR4 signaling, oxidative stress, epithelial barrier dysfunction, and dysregulated mucosal immunity. These findings highlight their potential roles in the prevention and attenuation of NEC. Future therapeutic strategies should focus on appropriate dosing, developmental considerations, and combination approaches to maximize efficacy while minimizing risk in preterm infants.

## 1 Introduction

Necrotizing enterocolitis (NEC) is a severe gastrointestinal disorder that primary affects preterm and low-body-weight infants, particularly those receiving eternal feeding (1). It is characterized by ischemic necrosis of both the small and large intestines. As a result, enteric bacteria can translocate into the bloodstream, frequently leading to severe sepsis and death (2–7). Reported mortality rates range from 15 to 45% (3, 8, 9). Infants who survive often experience long-term complications, including short-bowel syndrome, growth restriction, and neurodevelopmental impairment (10, 11). The pathogenesis of NEC is multifactorial and remains incompletely understood. Current evidence suggests that NEC develops through a combination of intestinal dysbiosis characterized by reduced microbial diversity, exaggerated mucosal immune responses, and, in some cases, genetic susceptibility (12–15).

One of the key events in NEC development is extensive intestinal epithelial cell death, which disrupts barrier integrity and increases intestinal permeability. NEC has been strongly associated with Gram-negative bacteria, and Toll-like receptor 4 (TLR4), a key innate immune receptor, is highly expressed in the premature intestine. Lipopolysaccharide (LPS), a major component of the outer membrane of Gram-negative bacteria, is recognized by TLR4 and its co-receptor MD-2 on host immune cells. This interaction activates nuclear factor-kappa B (NF-κB) signaling, leading to the production of pro-inflammatory cytokines that initiate and amplify immune responses.

Among helper T-cell subsets, Th17 and Th1 cells play important roles in intestinal immunity. Th17 cells are particularly involved in host defense against extracellular bacteria and fungi through the secretion of interleukin-17 (IL-17) and other cytokines that recruit neutrophils and additional immune cells to sites of infection (16). However, excessive Th17 responses contribute to NEC pathogenesis by promoting inflammation, disrupting epithelial barrier function, and exacerbating tissue injury (17, 18).

Current treatment strategies for NEC are largely supportive. In the early stages of disease, management includes cessation of enteral feeding, broad-spectrum antibiotics, and fluid and inotropic support. In advanced cases, surgical intervention, including resection of necrotic bowel and abdominal drainage, may be required. Several emerging therapeutic approaches, including TLR4 inhibitors (19, 20), aryl hydrocarbon receptor agonists (21, 22), stem cell therapies (23), synthetic amniotic fluid (24), breast milk exosomes (25, 26), anti-cytokine therapy, growth factor therapy (27) etc. have been considered as drug candidate compounds, but those are still in development.

Vitamins play important roles in maintaining intestinal health by supporting epithelial barrier function, regulating immune responses, and influencing the composition of the gut microbiota. Deficiencies in several vitamins have been associated with increased inflammation, whereas adequate vitamin intake, particularly of vitamins A, C, and D, has been linked to reduced inflammatory responses and improved microbial balance (28, 29). These observations suggest that vitamins may have therapeutic relevance in NEC, a disease in which dysbiosis, inflammation, and epithelial barrier dysfunction are central features (30). Intestinal dysbiosis is increasingly recognized as a central contributor to NEC pathogenesis. In addition to influencing immune maturation and epithelial barrier function, the gut microbiota participates in vitamin metabolism and biosynthesis, while vitamin status can shape microbial composition and function. These bidirectional interactions suggest that vitamins may influence NEC not only through direct effects on host tissues but also through microbiota-mediated mechanisms.

Interest in the relationship between vitamins and NEC has therefore increased in recent years. However, although several physiological and clinical studies have examined the role of vitamins in NEC, the underlying molecular mechanisms remain incompletely understood. In this review, we summarize both direct and indirect evidence regarding the roles of vitamins in NEC pathogenesis and protection. We focus on four major areas: regulation of immune responses, modulation of LPS-TLR4-NF-κB signaling, maintenance of epithelial barrier integrity, and enhancement of immunoglobulin A (IgA)-mediated mucosal defense. We also discuss the biochemical and molecular mechanisms through which specific vitamins may attenuate intestinal inflammation and tissue injury associated with NEC.

## 2 Biological functions of vitamins

### 2.1 Vitamin A

Vitamin A exists in two important forms: preformed retinoids (retinol and its derivatives) and provitamin A carotenoids (primarily *β*-carotene) (31). In addition to its well-established role in vision, vitamin A is essential for growth, reproduction, cell division, and immune function (32).

The metabolite 11-cis-retinal serves as the photoreactive component of rhodopsin and is critical for vision (33). Most other biological functions of vitamin A, including development, epithelial differentiation, bone formation, reproduction, and immune regulation, are mediated by its active metabolite, retinoic acid.

Vitamin A also exhibits antioxidant activity (34). And has been investigated in several inflammatory disorders, including acne vulgaris, bronchopulmonary dysplasia, and certain premalignant and malignant conditions.

Within the gut-associated lymphoid tissue (GALT), vitamin A plays an important role in maintaining intestinal mucosal integrity, promoting epithelial differentiation, and regulating inflammatory responses (35).

Retinoic acid, which is abundant in the intestinal environment, is increasingly recognized as a key regulator of mucosal immunity. It promotes gut-homing properties in both CD4 + and CD8 + T lymphocytes (36). In addition, retinoic acid produced by intestinal dendritic cells (DCs) induces gut tropism in B cells and, together with IL-5 or IL-6, enhances IgA production (37).

### 2.2 Vitamin D

Vitamin D is synthesized in the skin when 7-dehydrocholesterol in the epidermis is exposed to ultraviolet B (UVB) radiation, resulting in the production of cholecalciferol (vitamin D_3_) (38). In addition to cutaneous synthesis, vitamin D_3_ can be obtained from dietary sources such as egg yolks and oily fish, whereas ergocalciferol (vitamin D_2_) is found primarily in mushrooms (39, 40).

As a fat-soluble vitamin, vitamin D is essential for calcium and phosphorus homeostasis, bone health, and numerous physiological processes. Vitamin D_2_ is mainly derived from plant sources, whereas vitamin D3 is derived primarily from animal sources.

Its biologically active metabolite, calcitriol, exerts its effects through binding to the vitamin D receptor (VDR), a nuclear receptor expressed in many target cells. Beyond its classical role in bone metabolism, vitamin D has been implicated in immune regulation, inflammation control, and protection against several chronic diseases. Epidemiological studies have associated low vitamin D status with increased susceptibility to autoimmune and chronic infectious diseases (41, 42).

Vitamin D is now recognized as an important immunomodulatory hormone with functions extending beyond calcium and bone metabolism. VDR is expressed in a wide range of immune cells, including macrophages, dendritic cells, B cells, and T lymphocytes, many of which can locally synthesize the active metabolite 1,25-dihydroxyvitamin D_3_ [1,25-(OH)₂D₃]. Through autocrine and paracrine signaling, vitamin D regulates both innate and adaptive immune responses and contributes to immune homeostasis (43, 44). Although *in vitro* experimental findings consistently support protective immunological roles of vitamin D, clinical supplementation trials in humans have produced variable results, likely due to differences in dosage, metabolite selection, timing, and treatment duration (44).

### 2.3 Vitamin C and vitamin E

Vitamin C (L-ascorbic acid) is an essential water-soluble micronutrient that humans must obtain through the diet because it cannot be synthesized endogenously. It functions as a potent antioxidant and an enzymatic cofactor involved in numerous metabolic and physiological processes. Vitamin C plays a central role in collagen biosynthesis by supporting hydroxylase and oxygenase enzymes through its reducing activity, thereby promoting proper collagen formation and tissue integrity (45, 46). In addition, it supports both innate and adaptive immunity by maintaining epithelial barrier integrity, enhancing antioxidant defenses, and promoting the activity of immune cells, including neutrophils, macrophages, B cells, and T cells. Vitamin C enhances neutrophil chemotaxis, phagocytosis, reactive oxygen species generation, and microbial killing, while facilitating the clearance of apoptotic neutrophils to reduce tissue damage (47).

Vitamin E (tocopherol) is an essential antioxidant that protects cellular membranes, particularly those of immune cells, from oxidative damage. It plays a critical role in maintaining immune function, and deficiency impairs host defense mechanisms, including resistance to infection, delayed-type hypersensitivity responses, and antibody production. Studies in both humans and animal models have shown that vitamin E supplementation at levels above the recommended dietary allowance can enhance humoral and cell-mediated immune responses and improve resistance to infectious diseases (48–51). As a lipid-soluble chain-breaking antioxidant, vitamin E protects against oxidative membrane damage (52, 53), regulates intestinal microbiota composition, and suppresses inflammatory signaling pathways (54). Its metabolite δTE-13′-carboxychromanol (δTE-13′) has been associated with increased *Lactococcus* and *Bacteroides* populations, alongside reducing inflammatory mediators (55).

### 2.4 Vitamin B_12_ and folate

Vitamins, especially folate (56), vitamin B_12_ (57), and vitamin B_6_ (58), play central roles in DNA methylation by supplying and processing the one-carbon units required for the synthesis of S-adenosylmethionine (SAM), the universal methyl donor. Folate provides methyl groups for the remethylation of homocysteine, while vitamin B_12_ serves as an essential cofactor in this reaction, and vitamin B_6_ supports upstream folate metabolism. Deficiency of these vitamins reduces SAM availability, leading to global and gene-specific hypomethylation, chromosomal instability, and increased disease susceptibility. Conversely, adequate intake of B vitamins helps maintain normal DNA methylation patterns and may contribute to protection against NEC. Other micronutrients, including vitamins A, C, D, and E, may also influence the epigenetic landscape through effects on chromatin regulation, DNA repair, and oxidative stress.

Folate (vitamin B_9_), a key component of one-carbon metabolism, is required for DNA synthesis, methylation reactions, and immune function. Folate can influence inflammatory responses through its roles in DNA methylation and nucleotide synthesis, although these effects may vary depending on the timing, dosage, and physiological context of supplementation (59–61). Recent studies have linked NEC with alterations in DNA methylation patterns (62–67). Global hypermethylation is evidenced in NEC newborn intestinal tissues, especially outside promoters and CpG islands (63–65). Transcriptional dysregulation of genes involved in immune responses and epithelial function accompanies these alterations (63). However, it is unclear how vitamin B_12_ and folate have an impact on NEC through DNA methylation, as the DNA methylation status on of each gene in each cell type is more important than the global DNA methylation status.

### 2.5 Vitamin K

Vitamin K is a fat-soluble vitamin that plays essential roles in coagulation, bone metabolism, and vascular integrity. Emerging evidence also suggests a potential role for vitamin K in NEC pathogenesis and protection. In addition to serving as a cofactor for *γ*-glutamyl carboxylase and other vitamin K-dependent proteins, vitamin K exhibits anti-inflammatory properties through inhibition of NF-κB signaling and antioxidant effects through suppression of reactive oxygen species generation (68).

Within cells, vitamin K functions through a cyclic redox process known as the vitamin K cycle (69). First, vitamin K is reduced to its active form, vitamin K hydroquinone (quinol), by the enzyme vitamin K epoxide reductase (VKOR) (70). Vitamin K hydroquinone then serves as a cofactor for *γ*-glutamyl carboxylase, also known as the vitamin K-dependent carboxylase (71), which catalyzes the conversion of glutamate residues to γ-carboxyglutamate. This reaction is coupled to the oxidation of vitamin K hydroquinone to vitamin K epoxide. Subsequently, vitamin K epoxide is reduced back to vitamin K by VKOR, thereby completing the cycle. The coordinated reduction, oxidation, and carboxylation reactions collectively constitute the vitamin K cycle.

Clinically significant vitamin K deficiency is uncommon in humans, partly because vitamin K2 is continuously recycled through the vitamin K cycle within cells (72).

**Figure 1:**
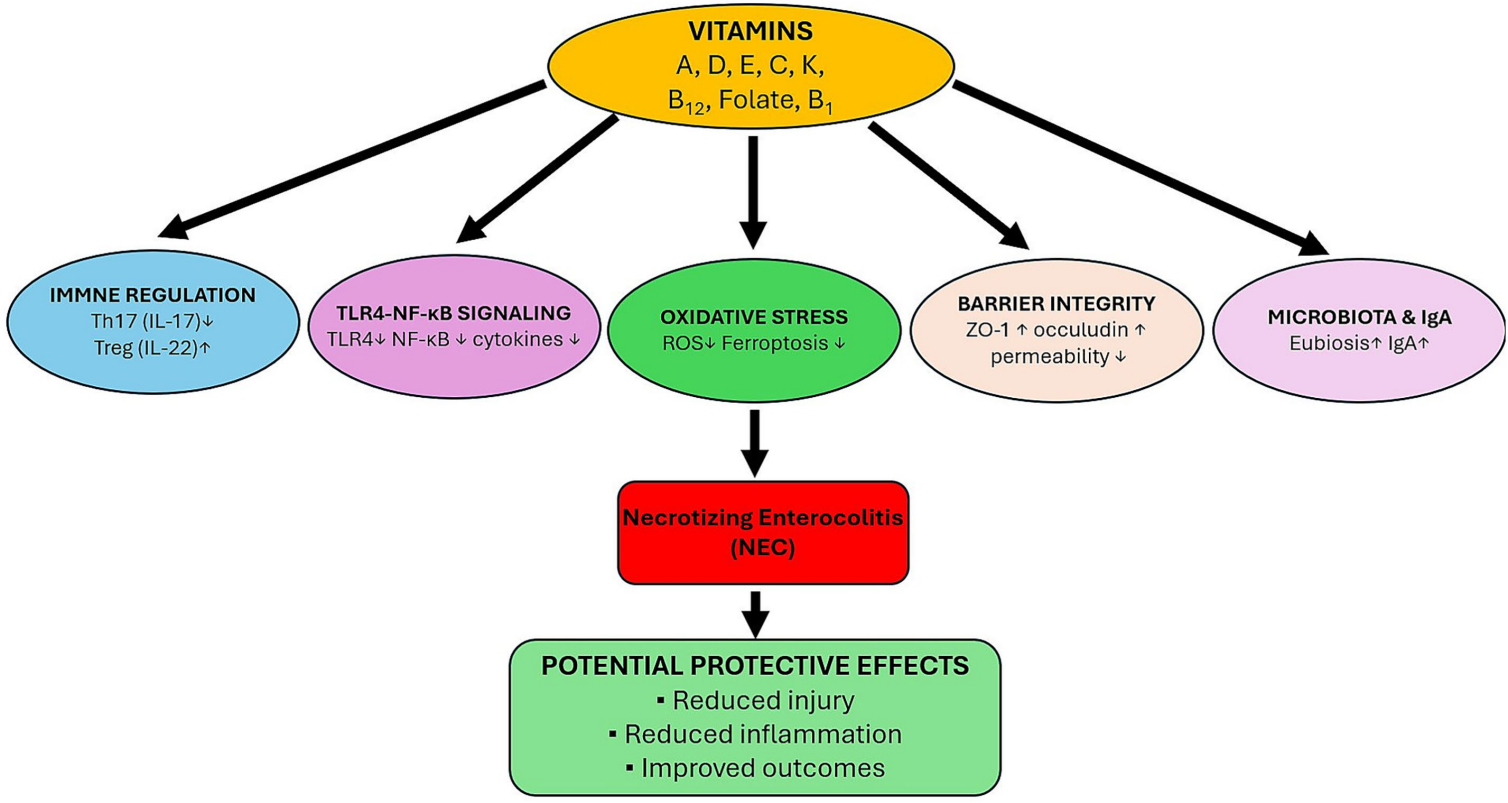
Proposed mechanisms through which vitamins influence NEC pathogenesis. Vitamins modulate key pathways involved in NEC, including immune regulation, TLR4-NF-κB signaling, oxidative stress, epithelial barrier integrity, and microbiota-mediated mucosal immunity. Through these mechanisms, vitamins may reduce intestinal injury and inflammation, preserve barrier function, and attenuate NEC severity.

Collectively, these vitamins influence key pathways involved in NEC pathogenesis, including immune regulation, epithelial barrier integrity, oxidative stress, and microbiota-mediated mucosal immunity (Figure 1).

## 3 Immune regulation by vitamins

### 3.1 Vitamin A

Several studies have investigated the possible protective role of vitamin A in relation to NEC. Vitamin A reduces intestinal injury in a mouse NEC *in vivo* model (73) and rat NEC *in vivo* and *in vitro* models (74, 75). It seems that the level of vitamin A inversely correlated to severity in NEC patients (73, 76). On the other hand, Rakshasbhuvankar *et al*. reported that vitamin A can promote NEC (77). There is a possibility that different doses of Vitamin A attenuate or promote NEC. Vitamin A may influence NEC through the following mechanisms.

Retinoic acid-related orphan receptor gamma t (RORγt) is a critical transcription factor for Th17 cell generation, maintenance, and function, as well as controlling the inflammatory response initiated by these cells (78, 79). NEC is not merely an inflammatory disease. NEC can be a RORγt-centered immune disorder in the premature intestine. RORγt is excessively activated in NEC and drives Th17 expansion and IL-17 production (18, 80).

It is also understood that NEC development reduces anti-inflammatory FOXP3^+^ Tregs to mice and humans’ premature small intestine, which can be reversed experimentally in mice by administration of all-trans retinoic acid (ATRA) (81).

Vitamin A, through retinoic acid, leads to lesser Th17 cell generation and inflammation by inhibiting RORγt expression through increasing TGF-*β*-mediated Smad3 signaling and inhibiting IL-6 and IL-23 receptor expression (82–84) and stimulates Treg differentiation through increased FoxP3 expression (83), which inhibits inflammation.

A number of studies have shown retinoic acid to mediate bidirectional T helper cell 17 (Th17) and regulatory T (Treg) cell differentiation, in which retinoic acid suppresses Th17 cell differentiation (85–89), while Treg cell induction by mucosal DCs is heavily dependent on retinoic acid and transforming growth factor-*β* (TGF-β) (90). Uematsu *et al*. recently reported that a low amount of retinoic acid seems to be necessary for Th17 cell differentiation (91). Thus, it is highly likely that retinoic acid modulates GALT homeostasis.

Retinoic acid in the immune system exerts immunomodulatory effects such as blocking activation-induced thymocyte and T cell apoptosis (92), blocking Th1 differentiation and promoting Th2 differentiation in inducing helper T cell differentiation (93, 94). On the other hand, it has been discovered that under a state of vitamin A deficiency, the production of the Th1 cytokine IFN-*γ* is significantly augmented, while Th2 function is dysfunctional (95).

### 3.2 Vitamin D

Vitamin D, through its active metabolite 1,25-dihydroxyvitamin D, controls the immune response via the VDR, expressed in T cells, dendritic cells, and intestinal epithelial cells (96–98). VDR signaling promotes differentiation and function of Tregs and suppresses Th1 and Th17 responses (99–101). In experimental NEC, epithelial integrity is preserved by vitamin D supplementation, tight junction protein expression is enhanced, and proinflammatory cytokine secretion, i.e., IL-17 (102–104), is limited. In addition, vitamin D modulates the intestinal microbiota by inducing eubiosis, which indirectly preserves Treg homeostasis and limits IL-17-driven inflammation (28, 105–107).

Mechanistically, vitamin D suppresses IL-17 production by conventional Th17 cells and CCR9^+^ IL-17-producing Tregs, skewing the immune balance toward regulatory phenotypes and reducing NEC severity (17, 108, 109). Concurrently, vitamin D enhances IL-22 production by ILC3s and other mucosal lymphocytes, which promotes epithelial regeneration and antimicrobial defense (110–112). These results are consistent with clinical results showing that vitamin D sufficiency is linked to reduced proinflammatory cytokines, improved intestinal barrier function, and reduced gut inflammation severity (100, 101, 113). Importantly, VDR control has also been shown to be effective in non-calcemic vitamin D analogues, offering further therapeutic options for NEC without hypercalcemia risk (114, 115).

### 3.3 Vitamin C and vitamin E

Preclinical studies indicate that dysregulation of oxidative stress in NEC causes Treg ferroptosis, reducing immunosuppressive function, and exacerbating inflammation and that vitamins C and E act synergistically to preserve Treg activity and limit intestinal injury (52, 116). Vitamin C is also known to increase the activity of TET enzymes and supports the demethylation of the conserved non-coding sequence 2 locus of FOXP3, which helps maintain the stability of Tregs (117).

Oxidative stress resulting from an imbalance between endogenous antioxidant defense and reactive oxygen species is a principal driving force behind NEC (118, 119). Vitamins C and vitamin E are part of the system, not only as antioxidants but also as immune-modulating drugs.

Vitamin E, in experimental NEC, protects Tregs from ferroptosis, which preserves their immunosuppressive activity and capping tissue damage (116).

A study using mice demonstrated that reduced GPX4 expression induces ferroptosis in Treg cells, contributing to excessive intestinal inflammation and tissue damage in NEC (116). GPX4 is the master regulator and primary endogenous inhibitor of ferroptosis. It functions as the ultimate defense barrier that prevents cells from undergoing this iron-dependent, lipid peroxidation-driven form of programmed cell death (120). GPX4-deficient Treg cells showed impaired immunosuppressive function and increased susceptibility to ferroptosis. Vitamin E supplementation inhibited Treg cell ferroptosis by enhancing GPX4 expression, thereby restoring Treg function and alleviating intestinal injury and inflammation in NEC.

Another study in premature infants found that deficiencies in vitamins A and E were associated with impaired immune function and increased NEC risk (121). Supplementation with vitamins A and E significantly improved T lymphocyte profiles and reduced NEC incidence, with combined supplementation showing the greatest protective effect. Higher vitamin A and E levels correlated with enhanced immune function and lower NEC occurrence, suggesting that these vitamins may help prevent NEC by strengthening neonatal immune responses.

### 3.4 Folate

Hyperhomocysteinemia (HHcy), the common outcome of folate inadequacy, has been shown to augment CD4^+^ T cell differentiation into proinflammatory Th17 cells with increased secretion of IL-17 and downstream signaling favoring degradation of tight junction integrity (122). Suppression of Th17 differentiation by folate supplementation confers protective an effect in maintaining epithelial barrier function.

This dialogue also holds in systemic immune modulation. In NEC, dysfunction and frequency of Treg cells have been described in neonates, leading to hyperinflammation via IL-17 and enhanced apoptosis of the epithelium (123). HHcy, which is usually associated with disturbed folate metabolism, could be employed to enhance this Th17/Treg imbalance even further, further compromising tight junctions and enhancing intestinal permeability even further. These dysregulation of immunity are depicted in mouse and human models of NEC, including Th17 polarization, IL-17 production, and disruption of tight junction, all of which are dependent on TLR4 (17).

Folate’s availability also controls intestinal inflammation by microbiota-mediated mechanisms. Colitis models exhibit that supplementation of folate in the colon, by microbes, is established to enhance methylation reactions and suppress IL-17-NF-κB-mediated inflammatory pathways (124). These results describe a mechanistic interaction among microbiota community structure, folate-dependent methylation, and maintenance of epithelial barrier integrity. Further, maternal one-carbon metabolic dysfunction like folate and vitamin B_12_ deficiency likewise causes HHcy and IL-17/IL-10 impaired signaling that can influence neonatal intestinal barrier formation and susceptibility to NEC (125).

## 4 Vitamins and TLR4 signaling in intestinal homeostasis and inflammation

### 4.1 Vitamin A

TLR4 is a key player as a mediator of innate immunity in the gut through recognition of LPS and activation of downstream NF-κB-dependent proinflammatory signaling. Dysregulated TLR4 signaling also causes breakdown of the epithelial barrier, inflammation of the intestine, and pathology such as NEC and inflammatory bowel diseases (IBD) (126, 127). Vitamin A, in the retinoic acid state, has been reported to modulate epithelial barrier function, the development of immune cells, and TLR4 signaling, implying its therapeutic utility in maintaining intestinal homeostasis (128, 129).

Retinoic acid induces the expression of TLR4 on epithelial cells of the intestine through retinoic acid receptor beta (RARβ), maintaining barrier function by upregulating tight junction molecules such as ZO-2 (128). *In vitro* and *in vivo* models indicate that retinoic acid therapy increases transepithelial electrical resistance and reduces permeability in epithelial monolayers, an effect mediated by RAR*β*-TLR4 interactions (128). On the other hand, vitamin A nutrition controls TLR4-mediated colonic mucosa-associated microbiota, and vitamin A deficiency alters microbiota composition in a TLR4-dependent manner (126). β-Carotene, a vitamin A precursor, also suppresses LPS-mediated TLR4 signaling and following NF-κB activation in colonic epithelial cells, resulting in reduced inflammatory cytokine production and enhanced tight expression of junction protein (e.g., occludin, claudin-1) (129).

Retinoic acid exerts deep anti-inflammatory activity in different epithelial tissues via regulation of TLR4/NF-κB pathways. In a TGEV-induced model of intestinal injury, ATRA suppressed proinflammatory cytokine release (IL-1β, IL-8, TNFα) but increased IL-10 and secretory IgA, and at the same time downregulated TLR3, TLR4, RIG-I, and NF-κB phosphorylation (127). Similar mechanisms existed in non-intestinal epithelial cells, including mammary epithelial cells, in which retinoic acid blocked LPS-induced TLR4 expression and NF-κB activity, reducing IL-1β production (130). These observations suggest that retinoic acid causes a general anti-inflammatory effect by modulating TLR4-mediated signaling pathways.

Besides retinoid activity of natural origin, vitamin A has also been utilized as a therapeutic delivery-targeting moiety to TLR4-expressing cells. Vitamin A-conjugated liposomes with the encapsulation of TLR4-shRNA have been utilized to efficiently silence TLR4 in hepatic and pancreatic stellate cells with the consequences of reduced NF-κB activation, reduced proinflammatory cytokine production, and fibrosis improvement (131–133). Such approaches leverage stellate cells’ high retinoid uptake potential and demonstrate the potential for vitamin A as a modulator and delivery vehicle to disrupt TLR4 signaling.

### 4.2 Vitamin D

New evidence suggests that vitamin D supplementation would reduce intestinal inflammation, improve epithelial barrier function, and perhaps reduce NEC severity (134–136).

Vitamin D achieves these actions by augmenting VDR expression, which in turn modulates downstream signaling pathways, such as repression of TLR4/MyD88/NF-κB activation (137, 138). These molecular pathways suggest an anti-inflammatory action of vitamin D against NEC-associated barrier dysfunction.

In both *in vivo* and *in vitro* models of NEC, VDR agonists such as EB1089 or vitamin D supplementation reduce the expression of TLR4, MyD88, and NF-κB and therefore reduce the inflammatory response (137–139). In mouse NEC models, vitamin D reduces tissue damage, inhibits apoptosis, and stimulates epithelial cell growth, effects partially mediated by ERK signaling activation and suppression of LPS-induced inflammatory response (134, 140). These findings establish the mechanistic link between vitamin D/VDR activity and TLR4-mediated intestinal injury in NEC.

Vitamin D also exerts positive effects on intestinal microbiota composition of which in NEC pathogenesis is of utmost relevance (139, 141, 142). Preterm gut dysbiosis accounts for increased LPS exposure and TLR4 activation. Preclinical studies show that 1,25(OH)₂D₃ increases the growth of commensal gut bacteria like *Muribaculaceae* and *Lachnospiraceae*, restores microbial diversity, and decreases systemic endotoxemia (139, 141). Vitamin D insufficiency is common in preterm and very low body weight (VLBW) infants and is associated with higher NEC, sepsis, and other morbidities (143–145). Although direct proof for vitamin D in the prevention of NEC in humans is scarce, preclinical research strongly supports its protective effect through modulation of TLR4 signaling, preservation of epithelial barrier function, and regulation of intestinal microbiota (134, 135, 137, 138). Future randomized clinical trials are warranted to determine the optimal dose, timing, and formulation of vitamin D in high-risk neonates.

### 4.3 Vitamin C and vitamin E

Vitamin C modulates inflammatory cytokine production in immune cells. In peripheral blood lymphocytes, vitamin C reduced LPS-induced production of the pro-inflammatory cytokines TNF-*α* and IFN-*γ* while enhancing anti-inflammatory IL-10 secretion, without affecting IL-1β levels (146). In contrast, treatment of peripheral blood monocytes with vitamin C and/or vitamin E enhanced LPS-stimulated TNF-α production but similarly showed no effect on IL-1β generation (147). These findings suggest that vitamin C exerts context-dependent immunomodulatory effects on inflammatory responses, although there is no direct evidence that vitamin C suppresses cytokine induction in NEC.

In NEC, however, the benefits of supplementation are balanced against potential risks. Retrospective analysis suggests oral vitamin E may increase the incidence of NEC in infants <1,250 g but intramuscular administration does not (148). Furthermore, pharmacologic serum levels of vitamin E were associated with heightened NEC and sepsis in VLBW infants on the premise of inhibited oxygen-dependent microbial killing, and oral or high-dose supplementation can interfere with vitamin K metabolism, increasing the risk of hemorrhage (148–150).

### 4.4 Folate

Feng et al. (151) demonstrated that folate significantly inhibits LPS-induced NO production, TNFα, and IL-1β in RAW264.7 macrophages, with decreased mRNA levels of iNOS, TNFα, and IL-1β. Folate acts mechanistically by inhibiting the phosphorylation of MAPKs and nuclear translocation of NF-κB p65, highlighting the ability of folate to interfere with key proinflammatory signaling events occurring in activated macrophages. Complementary studies in human THP-1 macrophages confirmed that folate, alone or together with other methyl donors, suppresses the expression and secretion of IL-1β and TNF but regulates transcription of chemokines such as CCL2 (60). These actions are accompanied by modifications in DNA methylation within cytokine loci, suggesting that epigenetic regulation could be part of the anti-inflammatory actions of folate.

### 4.5 Vitamin K

Using human and mouse macrophage-like cells, Ohsaki *et al*. demonstrated that multiple vitamin K analogues suppressed the expression of inflammatory cytokines triggered by LPS stimulation. Mechanistic analyses revealed that the anti-inflammatory activity was linked specifically to the 2-methyl-1,4-naphthoquinone ring structure of vitamin K, whereas the isoprenyl side chains alone lacked activity. Importantly, warfarin did not block these anti-inflammatory effects, indicating that the mechanism is independent of vitamin K-dependent *γ*-carboxylation and Gla protein synthesis. The study further showed that vitamin K₂ inhibited activation of the NF-κB pathway by suppressing phosphorylation of IKKα/*β*, key upstream regulators of NF-κB signaling. Consequently, NF-κB activation and downstream inflammatory cytokine production were markedly reduced after LPS exposure (152).

Vitamin K cycle allows for cyclical recycling of small quantities of vitamin K and therefore sustains the activation of vitamin K-dependent proteins such as clotting factors and protein S (PS), which is necessary for hemostatic equilibrium (68). PS, in turn, is an important anticoagulant in plasma and, by virtue of deficiency secondary to inflammatory stimuli such as LPS, can generate thrombotic tendencies, even exacerbate intestinal damage in NEC (153). Decreased PS expression is mediated through MEK/ERK and NF-κB activation. This process involves CD14 and TLR4, linking vitamin K-dependent mechanisms to innate immune signaling (153).

Clinical practice among preterm infants supports the necessity of adequate vitamin K prophylaxis at birth. Low and comparable NEC rates were observed in all groups in a randomized trial comparing intramuscular doses of 1.0 mg, 0.5 mg, and 0.3 mg of vitamin K_1_ in very preterm or VLBW neonates, proving that adequate vitamin K can be achieved through varying dosing regimens (154). Experimental evidence also suggests that vitamin K controls inflammatory signaling by suppressing TLR2 and TLR4, the central integration point of innate immune activation and gut inflammation (155). Beyond vascular and immunomodulatory actions, vitamin K also influences metabolic processes containing glutathione and *β*-alanine, possibly further controlling intestinal oxidative stress and inflammatory responses (156).

## 5 Vitamins and epithelial barrier integrity in NEC

### 5.1 Vitamin A

Vitamin A and its derivatives, including retinoic acid and all-trans retinoic acid (ATRA), play important roles in maintaining intestinal homeostasis through regulation of epithelial barrier function, immune responses, and gut microbiota composition (73, 157, 158). Vitamin A enhances the expression of tight-junction proteins such as ZO-1, occludin, and claudin-1, which are essential for maintaining barrier integrity and preventing bacterial translocation. Experimental studies have shown that vitamin A supplementation restores tight-junction protein expression, increases transepithelial electrical resistance, and reverses LPS-induced epithelial barrier injury (73, 157). In NEC, disruption of the intestinal epithelial barrier contributes to mucosal injury and excessive inflammation. Studies using neonatal rat and Caco-2 cell models have demonstrated that vitamin A and retinoic acid reduce inflammatory cytokine expression, improve tight-junction integrity, and protect against epithelial damage, suggesting direct barrier-protective effects (73, 158).

*β*-Carotene, a precursor of vitamin A that is abundant in breast milk, also protects intestinal epithelial cells from NEC-associated injury. In IEC-6 cells, β-carotene suppresses LPS-induced apoptosis and autophagy through activation of the PI3K/AKT/mTOR signaling pathway, highlighting the importance of intracellular signaling in epithelial barrier protection (75). In addition, ATRA reduces oxidative stress and inflammation in neonatal NEC models by increasing antioxidant enzyme activity and decreasing malondialdehyde and TNF-*α* levels, thereby preserving epithelial integrity (74). Moreover, ATRA induces expression of TGF-β2 in intestinal epithelial cells through activation of ATF2 by RhoA- and p38α- MAPK pathways, providing resilience to anti-inflammatory and protective barrier responses (159).

Modulation of the gut microbiota represents another mechanism through which vitamin A supports epithelial barrier function. In experimental NEC, vitamin A supplementation alters microbial composition by reducing potentially pathogenic taxa such as *Escherichia-Shigella* and increasing beneficial bacteria including Bacteroides, thereby attenuating inflammation and improving barrier integrity (73).

### 5.2 Vitamin D

Vitamin D is a key regulator of intestinal barrier function and tight-junction integrity. Its active metabolite, 1,25-dihydroxyvitamin D, acts through the vitamin D receptor (VDR), which is highly expressed in intestinal epithelial cells and regulates the transcription of numerous genes involved in barrier maintenance, inflammation, and cellular homeostasis (160–162). Experimental studies have demonstrated that vitamin D deficiency or VDR deletion disrupts tight junctions, increases intestinal permeability, and exacerbates inflammatory responses, highlighting the importance of intact vitamin D signaling in maintaining epithelial barrier integrity (163–165). Consistent with these findings, 1,25(OH)₂D₃ restores tight-junction expression, enhances transepithelial electrical resistance, and reduces permeability in LPS-treated intestinal epithelial cells (135).

Mechanistically, vitamin D maintains tight junction integrity through several pathways. Vitamin D has been shown *in vitro* and in animal studies to promote the expression of claudin-1, claudin-3, and claudin-5 barrier-forming claudins) and suppress claudin-2, and claudin-12 (pore-forming claudins), strengthening epithelial sealing (163, 166, 167). Epigenetic regulation also contributes to these effects, as vitamin D/VDR signaling inhibits histone deacetylase 11 binding to promoters of tight-junction genes, resulting in increased transcription of barrier-associated proteins (164). Furthermore, vitamin D interacts with the Notch and Wnt/*β*-catenin pathways to promote epithelial differentiation, tight-junction expression, and barrier function under inflammatory conditions (166, 168).

Vitamin D deficiency worsens disease severity in experimental NEC models by increasing intestinal permeability, disrupting tight junctions, and enhancing inflammation, whereas vitamin D supplementation or VDR activation attenuates these pathological changes (166, 168, 169). Additional evidence from stem cell-based studies demonstrates that restoration of barrier function through modulation of endoplasmic reticulum stress rescues tight-junction expression and reduces permeability, emphasizing the importance of epithelial integrity in NEC prevention (170). Translational and clinical studies further support the potential therapeutic value of vitamin D in diseases characterized by epithelial barrier dysfunction, including NEC and mucositis. Vitamin D suppresses NF-κB-mediated inflammation, preserves tight-junction protein expression, and reduces microbial translocation, thereby limiting intestinal injury (160, 171). Moreover, maternal vitamin D deficiency has been shown to impair offspring intestinal barrier function through reduced claudin-1 expression and suppression of Wnt/*β*-catenin signaling, suggesting that early-life vitamin D status may influence NEC susceptibility (168).

Collectively, these findings indicate that disruption of epithelial barrier integrity is a hallmark of NEC and that vitamin D/VDR signaling plays a central role in barrier preservation. Through regulation of tight-junction proteins, epigenetic mechanisms, and signaling pathways such as Notch and Wnt/β-catenin, vitamin D helps maintain intestinal epithelial integrity and may reduce NEC severity. These observations support further investigation of vitamin D supplementation and VDR-targeted therapies for the prevention and treatment of NEC.

## 6 Vitamins and IgA in mucosal immunity: implications for NEC

### 6.1 Vitamin A

Vitamin A is an important regulator of mucosal immunity, particularly through its effects on immunoglobulin A (IgA), the predominant antibody at mucosal surfaces. IgA limits microbial adherence, neutralizes pathogens, and helps maintain intestinal barrier integrity, all of which are critical in the context of NEC. In preterm infants, impaired IgA responses are associated with dysbiosis, excessive inflammation, and increased susceptibility to NEC, highlighting the importance of factors that promote IgA production, including vitamin A (172–175).

It has been discovered that under a state of vitamin A deficiency, IgA antibody production is prone to decrease (95). Similarly, vitamin A-deficient rats exhibit marked reductions in IgA + plasma cells within the ileal lamina propria, as well as decreased numbers of CD4 + T cells and activated CD25 + lymphocytes in Peyer’s patches (PP), which are essential for sustaining IgA responses (176). These findings show that vitamin A deficiency affects the cellular and humoral components of mucosal immunity, providing a mechanistic explanation for the increased morbidity and mortality from enteric infections reported in deficient populations.

Vitamin A also plays an important role in antigen-specific IgA responses. Retinoic acid, the active metabolite of vitamin A, promotes B-cell gut homing through induction of α4*β*7 and CCR9 expression, facilitates IgA class switching, and supports plasma cell survival in cooperation with TGF-β, IL-5, and IL-6 (177, 178). In IL-5 receptor-deficient mice, vitamin A supplementation failed to enhance IgA responses, highlighting the importance of Th2 cytokine signaling in mediating vitamin A-dependent mucosal immunity (178). In addition, vitamin A deficiency impairs IgA responses to mucosal pathogens and vaccines in both the respiratory and gastrointestinal tracts, effects that can be restored by retinoid supplementation (179–181).

Clinical and translational studies further support a role for vitamin A in IgA-mediated immunity relevant to NEC. Oral vitamin A supplementation enhanced IgA responses to porcine epidemic diarrhea virus infection in gilts, increasing IgA-secreting cells in serum and milk and improving lactogenic protection of nursing piglets (182). In human infants, maternal IgA in breast milk shapes the developing intestinal microbiota, suppresses expansion of disease-associated Enterobacteriaceae, and protects against NEC. In contrast, formula-fed infants and infants with reduced IgA exposure exhibit lower levels of IgA-coated bacteria and an increased risk of NEC (172, 173, 183). Collectively, these findings suggest that vitamin A supports IgA-mediated mucosal defense through multiple complementary mechanisms. Retinoic acid promotes differentiation of B cells into IgA-secreting plasma cells, enhances gut homing, and supports the maintenance of CD4 + T-cell populations within Peyer’s patches that facilitate IgA production. Conversely, vitamin A deficiency disrupts these processes, resulting in impaired IgA responses, increased susceptibility to microbial dysbiosis, and heightened intestinal inflammation. These observations provide a mechanistic basis for the potential protective effects of vitamin A against NEC.

### 6.2 Vitamin B_12_

Evidence linking vitamin B_12_ to IgA-mediated mucosal immunity is limited. Studies using human colostrum have examined interactions among secretory IgA, lactoferrin, and vitamin B_12_-binding proteins. Although vitamin B_12_-binding proteins have been proposed to inhibit the growth of vitamin B_12_-dependent bacteria, naturally occurring secretory IgA did not enhance the bacteriostatic activity of either lactoferrin or vitamin B_12_-binding proteins (184). These findings suggest that the protective effects of neonatal secretory IgA are largely independent of vitamin B12-mediated antimicrobial mechanisms. However, they also indicate that interactions between IgA and vitamin B12-related pathways may be more complex than previously appreciated.

### 6.3 Vitamin B_1_

Naïve IgM^+^ B cells in PP undergo class switching to become IgA^+^ B cells, which requires a vitamin B_1_-dependent TCA cycle to generate ATP (185). As these cells differentiate into IgA-producing plasma cells within the lamina propria, their metabolic profile shifts toward increased reliance on glycolysis while maintaining TCA cycle activity to support robust IgA synthesis (186). Although IgA-producing plasma cells are largely unaffected by dietary vitamin B_1_ deficiency, regression of PPs occurs because of impaired maintenance of naïve B-cell populations (187). Consequently, vitamin B_1_ deficiency reduces antigen-specific IgA responses to oral antigens and vaccines, as PPs are essential sites for the initiation of mucosal IgA responses (187). These findings demonstrate that dietary vitamin B_1_ maintains the metabolic programs supporting naïve B cell survival and the initiation of intestinal IgA immunity. Although no direct experimental data currently link vitamin B_1_ deficiency to NEC, impaired IgA responses could theoretically compromise mucosal defense in the neonatal intestine, a key factor in NEC susceptibility.

## 7 Absorption of vitamins

Vitamins are classified according to their solubility as either water-soluble or fat-soluble. Fat-soluble vitamins, including vitamins A, D, E, and K, are absorbed in specific regions of the small intestine. Vitamin A is primarily absorbed in the proximal jejunum, vitamin D in the distal jejunum, and vitamins E and K in the ileum (188). Efficient absorption of these vitamins depends on normal intestinal function; therefore, intestinal injury or dysfunction may impair vitamin uptake and contribute to deficiency.

Interactions among fat-soluble vitamins can also influence absorption. Competitive uptake has been reported among vitamins D, E, and K, whereas vitamin A may reduce the absorption of other fat-soluble vitamins. In contrast, vitamin E has been shown to enhance vitamin A absorption (188).

These interactions should be considered when designing vitamin supplementation strategies.

Vitamin C is absorbed throughout the small intestine, with the highest absorption occurring in the distal small intestine, particularly the ileum. This process is mediated primarily by sodium-dependent vitamin C transporters and hexose transporters (189).

Folate is absorbed mainly in the duodenum and jejunum, whereas vitamin B_12_ is absorbed in the ileum after binding to intrinsic factor (190). Infants with NEC frequently require surgical resection of the terminal ileum, placing them at increased risk of vitamin B_12_ malabsorption and subsequent deficiency (191). Long-term complications of NEC include short-bowel syndrome, intestinal strictures, and developmental impairment (192). Loss of functional ileal surface area may compromise vitamin B_12_ absorption and increase the risk of megaloblastic anemia (193–195).

Studies of vitamin B_12_ absorption also provide insights into the relationship between nutrient uptake and mucosal immunity. Simultaneous administration of free and intrinsic factor-bound vitamin B_12_ in patients with immunoglobulin deficiencies, including agammaglobulinemia, demonstrated that vitamin B_12_ absorption can occur even in the absence of normal immunoglobulin levels. However, mucosal IgA may contribute indirectly to nutrient handling by maintaining intestinal homeostasis and protecting the epithelial surface during nutrient absorption (196). These observations suggest that IgA may have a supportive, rather than essential, role in vitamin B_12_ homeostasis.

## 8 Conclusion

Vitamins influence several key processes involved in NEC pathogenesis and may serve as adjunctive therapeutic agents through multiple molecular pathways. Vitamins A, C, D, E, K, and folate influence several key processes involved in NEC, including TLR4 signaling, oxidative stress, epithelial barrier dysfunction, and impaired mucosal immunity. Collectively, current evidence suggests that these vitamins may contribute to the prevention and attenuation of intestinal injury in NEC (Table 1).

**Table 1:** Summary of the proposed mechanisms through which vitamins influence NEC pathogenesis.

Vitamin	Major mechanisms in NEC	Key molecular targets	Evidence strength	References
Vitamin A	Barrier protection, IgA, Treg induction	RORγt, FoxP3, TGF-β, IgA	Strong	(73, 78, 79, 81, 95, 129, 130, 157, 158, 176)
Vitamin D	Barrier protection, immune regulation	VDR, TLR4, NF-κB, IL-17	Strong	(99–101, 137–139, 161, 162)
Vitamins E/C	Treg stability, Oxidative stress reduction	GPX4, ferroptosis, TET, FOXP3	Moderate	(117, 146, 147, 197)
Folate	Methylation, inflammation	NF-κB, IL-17	Emerging	(122, 124, 151)
Vitamin K	Anti-inflammatory, coagulation	TLR4, NF-κB, Protein S	Emerging	(152)
Vitamin B_1_	IgA metabolism	TCA cycle	Limited	(185, 186)

However, most mechanistic evidence is derived from experimental studies, and clinical data remain limited. Future studies should focus on defining optimal dosing regimens, timing of administration, and developmental considerations in preterm infants. In addition, combination approaches targeting multiple pathways may offer greater therapeutic benefit than individual vitamin supplementation alone. Further clinical studies are needed to determine the safety and efficacy of vitamin-based interventions for the prevention and treatment of NEC.

## Glossary

Akt: protein kinase B (PKB)
ATRA: all-trans retinoic acid
ATF2: Activating Transcription Factor 2
CCL2: C-C motif chemokine ligand 2
CCR9: C-C motif chemokine receptor 9
CD4: cluster of differentiation 4
CD8: cluster of differentiation 8
CD14: cluster of differentiation 14
CD25: cluster of differentiation 25
CpG: 5′-cytosine nucleotide-phosphate-guanine nucleotide-3’
DC: dendric cells
DNA: deoxyribonucleic acid
DTH: delayed-type hypersensitivity
ERK: extracellular signal-regulated kinase
GALT: gut-associated lymphoid tissue
GPX4: glutathione peroxidase 4
HHcy: hyperhomocysteinemia
IBD: inflammatory bowel diseases
IEC-6: intestinal epithelial cell-6
IFN-*γ*: interferon-gamma
IgA: immunoglobulin A
IgM: immunoglobulin M
IL-1β: interleukin-1 beta
IL-5: interleukin-5
IL-6: interleukin-6
IL-8: interleukin-8
IL-10: interleukin-10
IL-17: interleukin-17
IL-22: interleukin-22
IL-23: interleukin-23
ILC: innate lymphoid cell
ISCs: intestinal stem cells
JNK: c-Jun N-terminal kinase
ATP: adenosine triphosphate
TCA cycle: tricarboxylic acid cycle
SPL: sphingosine-1-phosphate lyase
SVCT: sodium-dependent vitamin C transporter
Lgr5: Leucine-rich repeat-containing G-protein coupled receptor 5
LPS: lipopolysaccharides
MAPK: mitogen-activated protein kinase
MEK: mitogen-activated protein kinase
mTOR: mammalian target of rapamycin
MyD88: myeloid differentiation primary response 88
NEC: necrotizing enterocolitis
NF-κB: nuclear factor kappa-light-chain-enhancer of activated B cells
NO: nitric oxide
PI3K: phosphatidylinositol 3-kinase
PP: Peyer’s patches
PS: protein S
RAR*β*: retinoic acid receptor beta
RhoA: Ras homolog family member A
RIG-I: retinoic acid-inducible gene I
mRNA: messenger ribonucleic acid
shRNA: short hairpin ribonucleic acid
RORγt: retinoic acid-related orphan receptor gamma t
ROS: reactive oxygen species
SAM: S-adenosylmethionine
SOCS1: suppressor of cytokine signaling 1
SOCS3: suppressor of cytokine signaling 3
Smad3: mothers against decapentaplegic homolog 3
TGEV: transmissible gastroenteritis virus
TGF-β: transforming growth factor-beta
Th1: T helper cell 1
Th2: T helper cell 2
Th17: T helper cell 17
TLR2: Toll-like receptor 2
TLR3: Toll-like receptor 3
TLR4: Toll-like receptor 4
TNFα: tumor necrosis factor-alpha
Treg: regulatory T cell
UVB: ultraviolet B
VDR: vitamin D receptor
VKOR: vitamin K epoxide reductase
VLBW: very low body weight
Wnt: wingless-related integration site
ZO-1: zonula occludens-1
ZO-2: zonula occludens-2
δTE-13′: δTE-13′-carboxychromanol
